# Detection and genotyping of restriction fragment associated polymorphisms in polyploid crops with a pseudo-reference sequence: a case study in allotetraploid *Brassica napus*

**DOI:** 10.1186/1471-2164-14-346

**Published:** 2013-05-24

**Authors:** Xun Chen, Xuemin Li, Bing Zhang, Jinsong Xu, Zhikun Wu, Bo Wang, Haitao Li, Muhammad Younas, Lei Huang, Yingfeng Luo, Jiangsheng Wu, Songnian Hu, Kede Liu

**Affiliations:** 1 National Key Laboratory of Crop Genetic Improvement, Huazhong Agricultural University, Wuhan 430070, China; 2 Key Laboratory of Rapeseed Genetic Improvement, The Ministry of Agriculture, Wuhan, China; 3 Beijing Institute of Genomics, Chinese Academy of Science, Beijing, China

**Keywords:** Polyploid crops, *Brassica napus*, Pseudo-reference sequence, Single nucleotide polymorphism, Presence/absence variation

## Abstract

**Background:**

The presence of homoeologous sequences and absence of a reference genome sequence make discovery and genotyping of single nucleotide polymorphisms (SNPs) more challenging in polyploid crops.

**Results:**

To address this challenge, we constructed reduced representation libraries (RRLs) for two *Brassica napus* inbred lines and their 91 doubled haploid (DH) progenies using a modified ddRADseq technique. A bioinformatics pipeline termed RFAPtools was developed to discover and genotype SNPs and presence/absence variations (PAVs). Using this pipeline, a pseudo-reference sequence (PRF) containing 180,991 sequence tags was constructed. By aligning sequence reads to the pseudo-reference sequence, allelic SNPs as well as PAVs were identified and genotyped with RFAPtools. Two parallel linkage maps, one SNP bin map containing 8,780 SNP loci and one PAV linkage map containing 12,423 dominant loci, were constructed. By aligning marker sequences to *B*. *rapa* sequence scaffolds, whose genome is available, we assigned 44 unassembled sequence scaffolds comprising 8.15 Mb onto the *B*. *rapa* chromosomes, and also identified 14 instances of misassembly and eight instances of mis-ordering sequence scaffolds.

**Conclusions:**

These results indicate that the modified ddRADseq approach is a cost-effective and simple method to genotype tens of thousands SNPs and PAV markers in a polyploidy plant species. The results also demonstrated that RFAPtools developed in this study are powerful to mine allelic SNPs from homoeologous sequences in polyploids, therefore they are generally applicable in either diploid or polyploid species with or without a reference genome sequence.

## Background

Single nucleotide polymorphisms (SNPs) are the most frequent polymorphism in the genomes of animals and plants. Next-generation sequencing (NGS) technologies are able to produce millions of short sequence reads in a high-throughput, cost-effective fashion and thus can be used for *de novo* sequencing and resequencing of genomes. When coupled with the barcoded multiplexed sequencing strategies [1], these technologies can be amended to carry out high-throughput SNP discovery and genotyping for a large number of samples simultaneously for whole-genome association studies to identify genes involved in complex genetic traits [2-6]. All these studies inevitably need a high-quality reference genome sequence for the alignment of genomic sequences of different individuals to call SNPs and genotypes. However, resequencing is currently not feasible for SNP discovery in the majority of crop species, especially those with large, polyploidy genomes such as oilseed rape, cotton, bread wheat and sugarcane, due to lack of a high-quality reference genome sequence and the existence of large amount of homoeologous sequences. In polyploid crops, for example *Brassica napus* L., the presence of two sub-genomes (A and C) makes SNP discovery and genotyping more challenging because homologous and homoeologous DNA sequences from the two sub-genomes have to be analyzed simultaneously. Two classes of DNA polymorphisms, inter-variety polymorphisms (allelic variations) and inter-homoeologue polymorphisms (IHPs), are usually mixed together. In addition, the original A and C genomes are already duplicated or triplicated as a result of ancestral polyploidization events [7-9], which further complicates the discovery and genotyping of SNPs in *B*. *napus*[10,11].

To overcome the complexity of genomes and improve genotyping through-put, approaches such as complexity reduction of polymorphic sequences (CRoPS), restriction-site-associated DNA sequencing (RADseq) and genotyping by sequencing (GBS) that coupled reduced representation libraries (RRLs) with NGS technologies have been developed [12-17] and extensively used to sequence a similar subset of restriction fragments from multiple individuals for accurate SNP discovery and genotyping at low cost [18]. Recently double digestion RADseq (ddRADseq) and double digestion GBS (ddGBS) were developed to simplify the procedure and reduce the cost of RRL construction [19,20]. At the same time, several bioinformatics approaches and tools have been developed for SNP discovery with reduced representation sequencing data in species without a reference genome sequence. In turkey, sequence reads from RRLs were *de novo* assembled into contigs, which subsequently served as a reference sequence to which all short reads derived from multiple individuals were mapped accurately [21]. Stacks and RADtools have been developed for SNP discovery and genotyping in several species [22,23]. But these tools were not specifically developed for SNP discovery in polyploid species such as oilseed rape and bread wheat, and thus could not effectively discriminate allelic variations from polymorphisms among homoeologous sequences. To date, no computational tool has the ability to unambiguously and efficiently discriminate SNPs from homoeologous sequence differences when both copies of the two homoeologous genomes are being sampled.

In this study, we employed a modified ddRADseq approach to sequence the reduced representation genomes of the two parents, ZY821 and No2127, and the BnaNZDH population derived from these two parents in deep coverage for SNP discovery and genotyping. We also developed a bioinformatics pipeline termed RFAPtools to unambiguously discriminate between SNPs versus differences of homoeologous sequences in polyploid crops such as oilseed rape without a reference genome sequence. A pseudo-reference sequence (PRF) was built using the sequence reads from the parents and their segregating DH population. By aligning sequence reads of each individual DH line to the PRF, both allelic SNP and presence/absence variations (PAVs) were discovered and genotyped.

## Results

### Distribution of *in silico* ddRAD tags in genome of *B*. *napus*

A modified ddRADseq protocol was used to construct RRLs for ZY821 and No2127, and their 91 DH progenies (Additional file 1: Figure S1). Genomic DNA was double-digested with restriction enzymes (RE) SacI and MseI, and fragments in a size range of 141–420 bp were recovered. Libraries from 12 different individuals tagged with 12 barcodes were pooled and sequenced on Illumina GAIIx or HiSeq2000 platforms. ZY821 and No2127 yielded 6.9 and 5.7 million PE reads respectively. The 91 DH individuals yielded a total of 212.3 million PE reads, ranging from 0.57 to 9.79 million reads in different DHs with an average of 2.33 million reads per DH line (Additional file 1: Figure S2).

**Figure 1:**
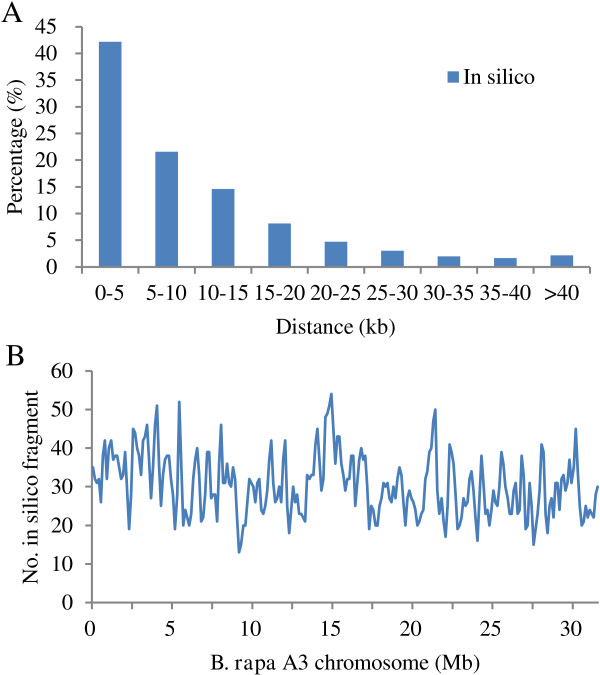
**Distribution of *in silico *tags along the *B. rapa *A03 reference genome sequence. A**. Distribution of physical distances between every two adjacent *in silico* restriction fragments in the size range of 141–420 bp. **B**. The distribution of *in silico* restriction fragments within the size range of 141–420 bp along the *B. rapa* A03 reference genome sequence within a sliding window of 250 kb.

To estimate the number of SacI-MseI fragments generated with ddRADseq in the *B*. *napus* genome, we did *in silico* double digestion of the *B*. *rapa* chromosome A03 (31.72 Mb in length) as a representative with the same restriction enzymes and obtained 3,904 SacI-MseI fragments within the size range of 141–420 bp. The distance between every two adjacent *in silico* sequence tags on the *B*. *rapa* A03 reference sequence was calculated. The majority (91.92%) of adjacent *in silico* tags were located within less than 25 kb, with 44.63% tags within 5 kb (0–5 kb) and 22.33% within 5–10 kb (Figure 1A). Only 1.89% adjacent tags were located 40 kb apart from each other. The average distance between two adjacent SacI-MseI fragments was about 8.1 kb. We further analyzed the distribution of SacI-MseI tags along the *B*. *rapa* A03 reference sequence in a sliding window of 250 kb (Figure 1B). The number of SacI-MseI tags varied from 13 to 55 in every 250 kb window. The distribution of *in silico* tags along the *B*. *rapa* A03 reference sequence indicated that the target fragments were evenly and randomly distributed in the *Brassica* genome.

The cumulative length of all *in silico* SacI-MseI fragments within the size range of 141–420 bp was 956,716 bp. Thus the genomic complexity should have been reduced by 33-fold (31,716,688 bp/956,716 bp). The total genome size of *B*. *napus* is estimated to be 1.2 Gb [24]. According to the *in silico* simulation and assuming that *B*. *rapa* and *B*. *napus* had similar GC content, genomic reduction with SacI and MseI double digestion should result in ~36 Mb of DNA or about 147,000 SacI-MseI fragments in the size range of 141–420 bp for sequencing, which is equivalent to about 3.0% of the *B*. *napus* genome. The above analyses suggested that the modified ddRADseq could significantly reduce the genome complexity of *B*. *napus* and also result in a considerably large number of target fragments for SNP discovery at the same time.

### SNP genotyping in DH population and linkage map construction

**Figure 2:**
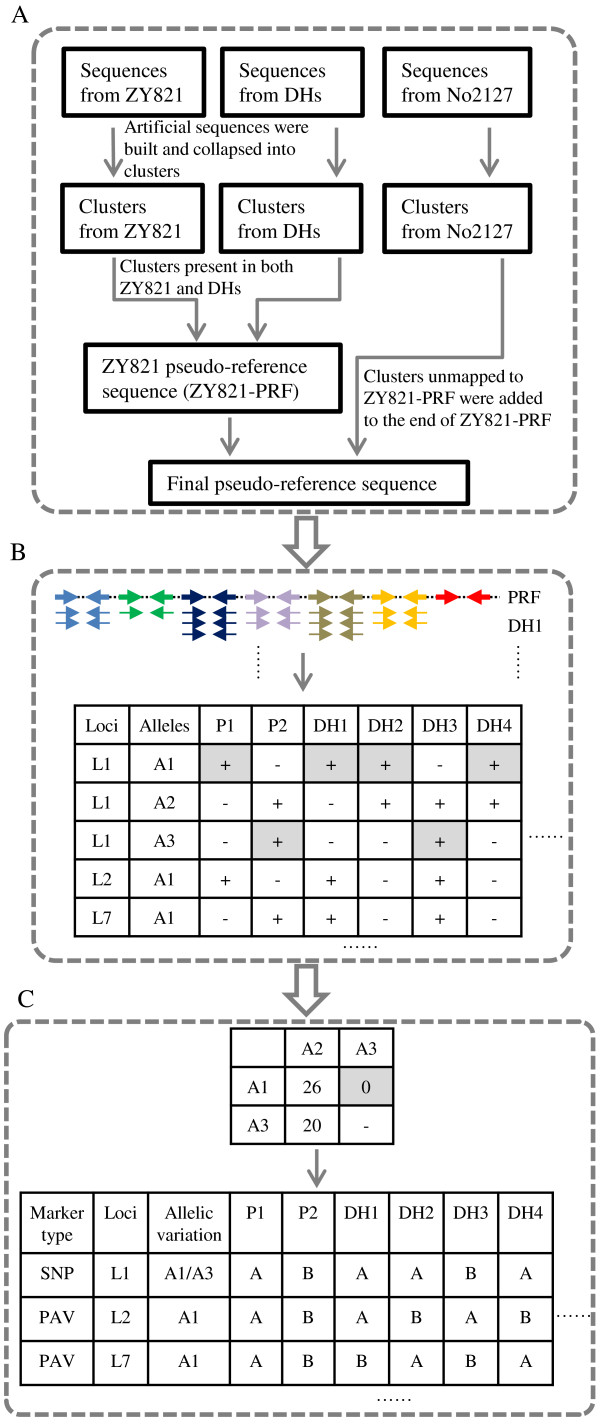
**The bioinformatics pipeline RFAPtools. A**. Assembly of pseudo-reference sequence. Pair-end sequence reads from ZY821, No2127 and pooled DHs were linked to form artificial sequence tags and collapsed into clusters. Then clusters present in both ZY821 and DHs were concatenated to assemble the ZY821 pseudo-reference sequence (ZY821-PRF). Thirdly, all clusters from No2127 were aligned to ZY821-PRF using SOAP with two nucleotide mismatches and those clusters that could not mapped were joined to the end of ZY821-PRF as clusters specific to No2127 to form the final pseudo-reference sequence. **B**. Discovery and genotyping of SNPs and PAVs. Sequencing reads of each individual were aligned to the PRF and all alleles at each locus were called as “+” indicating the presence of an allele and “-” indicating the absence of that allele in the two parents and 91 DH lines. **C**. Discrimination of allelic SNPs from homoeologous loci. A co-occurrence matrix of every two alleles from a complex locus with homoeologous sequences in the BnaNZDH population was made and allelic SNP and PAV loci were discriminated from homoeologous loci. Then genotypes of all individuals were scored for each locus.

SNP calling is usually performed by aligning short reads of one or more individuals to a high-quality reference genome using SOAP, MAQ or other software [6,25]. Currently, there is no such high-quality reference genome sequence and no computational tool available for *B*. *napus* to differentiate allelic SNPs from the abundant IHPs. To address these challenges, we developed a bioinformatics pipeline named RFAPtools that included three modules: assembly of a pseudo-reference sequence, SNP discovery and genotyping, and discrimination of allelic SNPs from homoeologous loci (Figure 2). Firstly, a pseudo-reference sequence was assembled using sequencing reads from the parents and DHs as described in the Method section. The pseudo-reference sequence contained a total of 180,991 sequence tags, including 22,670 sequence tags specific to No2127, which was very close to the above prediction.

**Figure 3:**
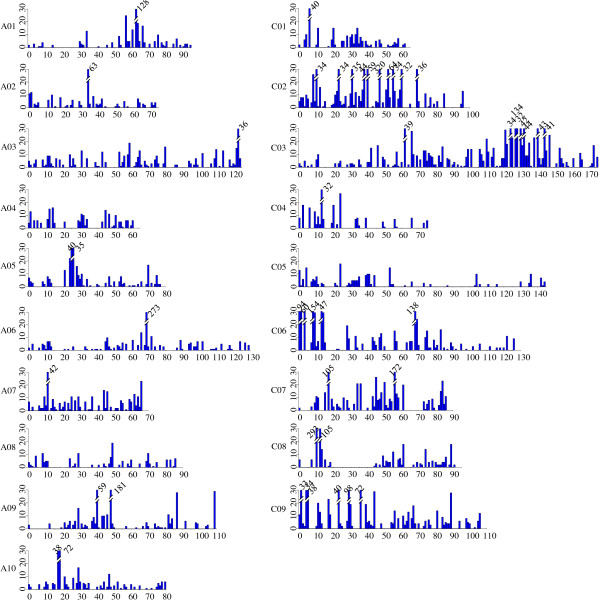
**The SNP bin map constructed using 91 DH individuals.** The X axis indicates the genetic distance (cM), and the Y axis indicates the number of SNP loci in each bin. The height of bar is maximized with 30 loci for each bin. The bins with more than 30 loci are marked individually with numbers on the bars.

Calling of SNP genotypes was done by aligning all sequence reads of the parents and individual DH to the pseudo-reference sequence using the SOAP software [26]. Each allele of a complex locus with mixed sequence tags was scored in each parent and DH individual using RFAPtools. Then a co-occurrence matrix of all alleles at the complex locus was made. Allelic variants were subsequently discriminated from homoeologous sequences based on the co-occurrence matrix with RFAPtools (Figure 2). Theoretically, a DH individual in a segregating DH population should be homozygous and a locus should only confer either allele from the two parents. With a threshold of 25% MMD (maximum missing data) in the DH population, a total of 9,210 simple SNP loci [27] containing 15,586 SNPs were recovered. SNPs distributed evenly across the PE reads, with a slightly decrease to the ends mainly due to the base quality declined (Additional file 1: Figure S3). Of these SNP loci, 7,468 (81.1%) had one unique allele from each parent, while the other 1,742 SNP loci were mined from 3 or more mixed homoeologous or paralogous sequences, which had been scored as hemi-SNPs in transcriptome sequencing [28]. All the 9,210 SNP loci, together with 47 single-locus SSR loci as anchors to assign linkage groups (LGs) to specific chromosomes, were used for linkage map construction. A linkage map comprising of 8,780 SNP loci containing 14,675 SNPs and 47 SSR loci was constructed using the MSTMap software [29]. The linkage map contained 895 bins with an average density of 9.86 loci per bin and covered a total length of 1,860.0 cM, with 3,116 loci mapped to the A sub-genome and 5,711 loci to the C sub-genome (Figure 3). A04 was the smallest LG that spanned 60.6 cM and comprised of 195 loci, and C03 was the largest LG that spanned 175.0 cM with 1,138 loci, which were consistent with previous linkage maps constructed with SSR markers [30]. All the information of the SNP linkage map was listed in Table 1.

**Table 1:** Summary of the SNP linkage map

**Linkage groups**	**Map length (cM)**	**No. bins**	**No. loci**	**Linkage groups**	**Map length (cM)**	**No. bins**	**No. loci**
A01	95.2	38	327(2)	C01	61.6	37	229(2)
A02	72.7	35	203(3)	C02	95.8	57	1023(2)
A03	124.4	65	397(3)	C03	175.0	101	1138(3)
A04	60.6	29	195(3)	C04	75.1	27	218(2)
A05	77.1	41	278(3)	C05	144.1	46	237(1)
A06	128.9	58	493(3)	C06	125.5	55	883(3)
A07	66.0	43	296(2)	C07	86.0	45	662(1)
A08	86.2	34	165(3)	C08	90.6	38	591(3)
A09	109.3	46	476(2)	C09	105.6	57	730(3)
A10	80.3	43	286(3)				
A genome	900.7	432	3116(27)	C genome	959.3	463	5711(20)

The number in the parenthesis represents the number of anchor SSR loci in this linkage group.

### PAV genotyping in DH population and linkage map construction

Two millions of sequencing reads were estimated to have a coverage depth of 10 folds at each locus on average based on the number of sequence tags in the pseudo-reference sequence. Thus we selected a subset of 45 DH lines, of which each comprised of about 2.0 million sequencing reads for PAV genotype calling. All PAVs that were perfectly aligned to the pseudo-reference sequence and appeared more than 200 times in the 45 DH lines were called and transformed into genotypes for each individual. A total of 20,856 dominant PAV markers were identified.

To construct a linkage map with the dominant PAV markers, 984 anchor SNP loci with 10% MMD in the 45 DH lines were selected from the SNP linkage map to form a skeleton map. A linkage map contained 12,423 dominant PAV loci was constructed with these anchor SNP loci (Additional file 1: Figure S4). The linkage map contained 707 bins with an average of 17.6 PAV markers in each bin and covered a total length of 2,076.8 cM. Of the dominant markers, 5,128 were located in the A sub-genome and 7,295 in the C sub-genome of *B*. *napus*. Consistent with the SNP genetic map, the largest and smallest linkage groups were C03 (189.3 cM) and A04 (66.8 cM) respectively. All information of the PAV genetic map was listed in Additional file 2: Table S1.

### Validation of SNPs and genotypes

To investigate the authenticity of identified SNPs, we randomly selected 44 SNPs that created or destroyed recognition sites of REs (called as cleaved amplified polymorphic sequence or CAPS marker) for validation of single nucleotide variations. PCR primers were designed to amplify the restriction fragments containing the SNPs. PCR products from the two parents were digested with the corresponding REs. Of the 44 SNPs, 26 (59%) could be confirmed by digestion, while the other 18 did not yield the expected restriction fragments. We further sequenced PCR products of all 44 loci amplified from the two parents using the Sanger sequencing method to further confirm these SNPs. All the 26 confirmed SNPs had the expected nucleotide variations, while the remaining 18 SNPs were a mixture of the expected allelic variations and homoeologous sequences. We also surveyed the 91 DH lines to validate the called SNP genotypes using the above confirmed 26 SNPs. A total of 2,251 genotypes were generated with an accuracy of 99.33%, indicating that the genotypes called by aligning sequence reads to the pseudo-reference sequence were reliable. These results further revealed the complexity of *B*. *napus* genome, and also demonstrated the efficacy of RFAPtools in discriminating allelic SNPs from homoeologous loci.

### Alignment of the SNP and PAV linkage map to the *B*. *rapa* reference genome

**Figure 4:**
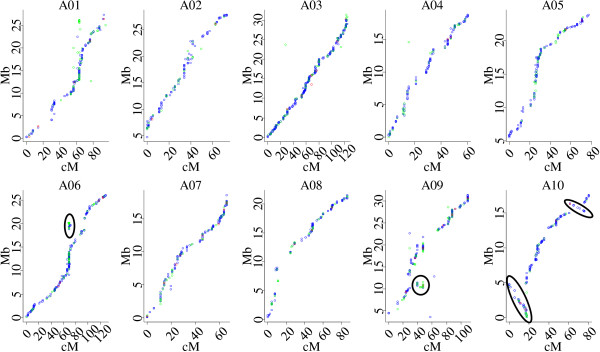
**Alignments between the SNP linkage map and the *B. rapa *reference genome sequence.** The X axis represents the genetic distance of each A genome linkage group in *B. napus*, and the Y axis represents the physical distance of reference sequence of each corresponding *B. rapa* chromosome. The green points represent the best hit (E value < =1e-10) of multiple paralogous loci in the *B. rapa* genome. The red points represent loci with only one end of the PE read having a unique position in the *B. rapa* genome. The blue points represent loci with both ends of the PE read having a unique position in the *B. rapa* genome. The oval circles indicate SNP loci that detected blocks of misassembly in the *B. rapa* reference genome sequence.

The sequences of all 3,116 SNP loci and 5,128 PAV loci mapped to LGs in the A sub-genome were aligned to the *B*. *rapa* reference genome sequence to validate the genetic linkage maps constructed in this study (Figure 4). If a locus was mapped to multiple paralogous positions in the *B*. *rapa* genome, only the location with the best hit was selected for collinearity analysis. Alignments indicated that all A sub-genome LGs of these two linkage maps had good collinearity with the *B*. *rapa* reference genome sequence except for minor inconsistencies, suggesting that the A sub-genome is retained relatively intact in *B*. *napus*[31] despite extensive genome rearrangements such as insertions and deletions that have occurred following hybridization of the A and C genomes [32,33]. The inconsistencies between the SNP linkage map and the *B*. *rapa* reference genome sequences such as regions on chromosome A02, A06 and A09 (Figure 4) may be caused by the existence of paralogous sequences, differences between *B*. *rapa* and *B*. *napus* genomes or misassembly of sequence scaffolds (Additional file 2: Table S2). For instance, the inconsistency involving 23 SNP markers around 69.4 cM on A06 was caused by possible misassembly of partial sequence within Scaffold000009 (4.67 Mb). The inconsistency from the 39.8 to 47.5 cM on A09 was caused by possible misassembly involved Scaffold000059, Scaffold000134, Scaffold000135 and Scaffold000145. An inversion from 61.6 to 75.9 cM involving 14 SNP markers was observed on A10, which corresponds to about 1.16 Mb of Scaffold000008 (Figure 4). Similar results were also observed between the A sub-genome of *B*. *napus* and the *B*. *rapa* A genome using markers on the PAV genetic map (Additional file 1: Figure S5).

### Mapping unassembled and misassembled scaffolds to the *B*. *rapa* chromosomes

High-density genetic linkage maps are often used to assign sequence scaffolds or contigs generated by whole genome shotgun sequencing to chromosomes. The genome of *B*. *rapa* was sequenced using the Solexa technology and 159 scaffolds comprised of 283.8 Mb draft genome sequence could be integrated to the *B*. *rapa* chromosomes [34], while 602 scaffolds larger than 2 kb could not be assigned to specific *B*. *rapa* chromosomes. Using the SNP and PAV high-density genetic maps, we were able to assign 44 unassembled scaffolds to specific *B*. *rapa* chromosomes (Additional file 2: Table S3). The assignment of a scaffold was declared only if two or more loci from the same or adjacent bins were mapped to the same scaffold and at least one SNP or PAV loci without paralogous sequences in *B*. *rapa* genome. Scaffold000100 (884.7 kb) was the largest and assigned to A06 by 17 SNP and 38 PAV loci. Scaffold000618 (3.9 kb) was the smallest and assigned to A06 by two adjacent PAV loci. In addition, we also identified 14 instances of and eight instances of misordering of sequence scaffolds (Additional file 2: Table S2). For instance, Scaffold000087, Scaffold000018, Scaffold000227 and Scaffold000136 which correspond to a chromosome region from 0 to 17.6 cM on A10, were mis-ordered as revealed by a run of 52 SNP and 61 PAV markers (Figure 4, Additional file 1: Figure S6), although we could not exclude the possibility of rearrangements in the A sub-genome of *B*. *napus.*

## Discussion

RRLs have been extensively used for sequencing a number of individuals in species with a reference genome sequence [16,35]. Methods for SNP discovery and genotyping generally require mapping sequencing reads to a fully sequenced genome from the same or a very closely related species. The unavailability of the whole genome sequence of the complex allotetraploid *B*. *napus* makes it still a challenge to determine genetic differences among individuals, which drastically limits this economically important oil crop to benefit from NGS technology. Therefore the development of a reference genome sequence is of high importance. In this study, we developed a pseudo-reference sequence using short reads both from the parents and the segregating DH individuals. Sequence tags detected in the two parents were included in the pseudo-reference sequence. With the pseudo-reference sequence, we could discover SNPs in the unsequenced *B*. *napus* using the reference-based SNP calling software such as SOAP that is solely developed for species having reference genome sequence. In addition, the pseudo-reference sequence constructed in this study could be used for any other studies such as genetic mapping and genome-wide association studies in *B*. *napus* using the same combination of restriction enzymes and library construction protocol.

In diploid species, it is relatively easy to call SNPs and genotypes because they do not have homoeologous sequences. But in polyploid species, the existence of homoeologous sequences makes it difficult to differentiate the allelic SNPs from the homoeologous and also paralogous sequence differences, because multiple sequence reads can align to the same genomic location or an individual sequence read can show significant alignment to multiple genomic locations. For these reasons, previous SNP identification methods have utilized only the subset of sequence reads that can be mapped uniquely to a reference [25] and discarded a significant percentage of otherwise mappable reads. *B*. *napus* is a recently formed polyploid originated from natural hybridization of the mesopolyploids *B*. *rapa* and *B*. *oleracea*, which contributed to the constituent A and C genomes, respectively. The ancestrally related segments of the progenitor A and C genomes are homoeologs and kept essentially intact in *B*. *napus*[31]. Therefore, polymorphisms detected from short reads in *B*. *napus* are a mixture of sequences at homoeologous loci, one corresponding to the A sub-genome homoeologue and the other to the C sub-genome homoeologue. Thus it is problematic to analyze allelic SNPs in *B*. *napus* as it is difficult to differentiate SNPs from the much more abundant inter-homoeolog polymorphisms (IHPs). In this study, we used two strategies in the RFAPtools to solve these problems. The first strategy was to separate the homoeologous sequences that differed with even only one nucleotide into distinct loci on the pseudo-reference sequence. The second strategy was to use the segregation data of the DH population to judge which two of the mixed alleles were allelic. In a segregating DH population with only homozygous genotypes, each individual should have two identical alleles from either parent. If two different alleles appeared in one individual, they should come from different homoeologous loci. Followed the segregation rule, we were able to distinguish allelic sequences from homoeologous sequences even just having one nucleotide variation between them using the integrated RFAPtools. Of the 9,210 SNP loci, 1,742 were mined from a mixture of homoeologous sequences. In contrast, only 19.3% SNPs could be identified in *B*. *napus* by aligning RNAseq reads to about 95,000 unigenes collected from the *Brassica* species [27,28], indicating that RFAPtools coupled with modified ddRADseq procedure could efficiently differentiate allelic SNPs from homoeologous sequence differences in polyploid species.

Many analyses of genetic variation using reduced representation sequencing only focused on discovery and genotyping SNPs [18,36]. However, structural variations are more than single-base-pair differences among individuals [37]. In whole genome resequencing, PAVs are identified to be structural variations caused by insertions or deletions in some regions of the genome, which could be scored as dominant markers. Here the PAVs could be caused either by insertions/deletions, or by mutations at RE recognition sites. Thus they may not be really absent in the genome but could be scored as dominant markers. As revealed by AFLP, PAVs associated with genome structural variation and mutations at RE recognition sites are highly abundant in *Brassica* genome [38]. In this study, 12,423 PAVs were scored as dominant markers in DH individuals having sequencing reads higher than 10 × coverage depth at each locus. The number of PAVs was much more than that of SNPs, which have not been identified in previous reduced representation sequencing reports [19,20].

*De novo* sequencing of the *B*. *napus* genome is undergoing with the NGS technologies. Assembly of short reads is a great challenge in such a complex genome and high-density sequence-based genetic maps can be a great help to assemble the sequence scaffolds to form integrated chromosomes. However, such a high-density linkage map that may aid in the ordering and anchoring of the sequence scaffolds or contigs is still lacking in the *Brassica* species. In this study, the two high-density linkage maps were constructed, which allowed us to assign 44 previously unassigned scaffolds to the *B*. *rapa* chromosomes and identify 14 possible misassembly and 8 possible misordering sequence scaffolds. Bancroft et al. identified 32 instances of misassembly of sequence scaffolds and a segmental rearrangement using a similar SNP bin map [28]. Therefore the two high-density linkage maps constructed in this study will be very useful for assignment and correction of sequence scaffolds in *de novo* sequencing of the A, C and AC genomes. Furthermore, these maps also provide a platform for maker-assisted selection breeding, map-based gene cloning and comparative genomic research in *Brassica* species.

## Conclusions

In this study, we constructed reduced representation libraries (RRLs) for two parents and their 91 DH progenies using a modified ddRADseq technique. A bioinformatics pipeline termed RFAPtools was developed for SNPs/PAVs discovering and genotyping in polyploid species. Using these tools, we constructed a SNP bin map containing 8,780 SNP loci and one PAV linkage map containing 12,423 dominant loci. Both linkage maps showed good collinearity with the *B*. *rapa* reference genome sequence. Using these linkage maps, we assigned 44 unassembled sequence scaffolds to the *B*. *rapa* chromosomes, and also identified 14 instances of possible misassembly and 8 possible instances of mis-ordered sequence scaffolds. These results suggested that modified ddRADseq procedure and the bioinformatics pipeline RFAPtools developed here is a powerful tool to mine allelic SNPs from homoeologous sequences, therefore they are generally applicable in either diploid or polyploid species with or without a reference genome sequence.

## Methods

### Plant materials and DNA extraction

Samples comprised of 91 DH lines from the BnaNZDH population along with its two parents, No2127 and ZY821. This BnaNZDH population has already been used to construct genetic linkage maps with microsatellite markers [30,39-41]. Total DNA was isolated from 250 mg young leaves of the parents and 91 DH lines.

### Designing adapters and the annealing

Two types of adaptors, MseAD and SacAD, compatible with the MseI and SacI cohesive ends, were designed for RRL construction. Each adaptor contains three parts, the common Illumina PE adapter sequence including sequencing primer, a 5-base barcode and complimentary sequence to the overhangs produced by the restriction enzymes. The top sequence of the MseAD and the bottom sequence of SacAD were 5′-phoshorylated. All adapter sequences including barcodes are listed in Additional file 2: Table S4. Adapters were created by mixing 1.0 nmol of each of the two corresponding top and bottom oligonucleotides in 20 μl water, heated at 94°C for 2 min, then ramped down to 65°C, 56°C, 37°C and 22°C by 0.5°C/s segmentally; and stopped at each temperature for 10 min.

### Library construction and illumina sequencing

About 200 ng of genomic DNA from each sample was double digested separately with 5 U of the restriction enzymes SacI and MseI (Fermentas) with 1 × restriction buffer in a reaction volume of 25 μl. The reaction mixture was first incubated at 37°C for 6 hr, then at 65°C for 90 min. The restriction fragments of each individual were then ligated to the SacAD and MseAD adaptors with unique barcode combinations to differentiate the samples. Ligation reaction was done in a reaction volume of 50 μl at 16°C for overnight with 25 pmol of SacAD and MseAD adaptors, and 50,000 Units of T4 DNA ligase. The restriction sites of both MseI and SacI were disrupted upon ligation to adaptors. In order to remove all residual recognition sites due to incomplete digestion or chimeric fragments generated by re-ligation, an additional double digestion with the same enzymes was performed. The final ligates of 12 individuals were pooled together to form a library and were separated on 2% agarose gel. Fragments in the size range between 220 and 500 bp, which corresponded to restriction fragments in the size range of 141–420 bp before adaptor ligation, were recovered from the gel and purified with the Qiagen gel purification kit. Each library was eluted with 25 μl H_2_O. Then each library was amplified in a 50 μl of volume with 50–100 ng of adaptor-ligated DNA fragments as template, 1× HF buffer, 3.5 mM MgCl_2_, 0.4 mM dNTPs, 1 U iProof polymerase (Bio-Rad), 5 pmol of two overhang primers (PF: ;5- AATGATACGGCGACCACCGAGATCTACACTCTTTCCCTACACGACGCTCTTCCGATCT and PR: 5-; CAAGCAGAAGACGGCATACGAGATCGGTCTCGGCATTCCTGCTGAACCGCTCTTCCGATCT). PCR amplification was performed as follows: 98°C for 2 min, followed by 11–13 cycles at 98°C for 30 s, 65°C for 30 s and 72°C for 15 s, and a final extension at 72°C for 5 min. PCR products were separated on 2% agarose gel and fragments in the size range of 270–550 bp were purified with the Qiagen gel purification kit and eluted with 25 μl H_2_O. The libraries were quantified using Qubit flourimeter (Invitrogen), Agilent 2100 (Agilent Technologies) and real-time quantitative PCR, then submitted for sequencing on Illumina GAIIx or HiSeq2000 platform. The clean data were then parsed into different individuals with exact match to the barcodes and remnant restriction sites at both ends. After trimming the barcode sequence, the first 70 nucleotides of each PE read were kept for further analysis.

### The RFAPtools pipeline

The RFAPtools include three modules: assembly of a pseudo-reference sequence, SNP discovery and genotyping and discrimination of allelic SNPs from homoeologous loci.

#### Assembly of a pseudo-reference sequence

Figure 2A depicts the five steps to make the pseudo-reference sequence (PRF) which was executed by the scripts including in prf_building.sh. Firstly, the first strand and the reverse complementary sequence of the second strand of each PE read were linked together to form a 200 bp artificial sequence tag by filling the unsequenced middle part with 60 cytosines (C). Then identical artificial sequence tags from all DH lines were collapsed into clusters. Meanwhile, the identical artificial sequence tags of each parent were also collapsed independently. A cluster that contained twenty or more artificial sequence tags from the DHs was treated as a unique tag to represent a SacI-MseI fragment in this study. Thirdly, in order to remove mitochondria and chloroplast sequences, all unique tags reads were subjected to BlastN search against the mitochondria and chloroplast database of oilseed rape with a cutoff E-value of 1e-15. Fourthly, the remaining unique tags that appeared in DHs and also in the ZY821 parent were tandemly linked to form the ZY821 pseudo-reference sequence (ZY821-PRF) by stuffing 100 guanines (G) to separate adjacent unique tags. All PE reads from No2127 were aligned to the ZY821-PRF using the SOAP software with at most two nucleotide mismatches on each strand of a PE read. The remaining unique tags corresponding to the unmapped PE reads were specific to No2127. Finally, the unique tags specific to No2127 were joined to the end of ZY821-PRF also by stuffing 100 guanines (G) between unique tags to form the final pseudo-reference sequence.

#### SNP discovery and genotyping

SNP and genotype calling were done by aligning all reads of the parents and each DH line to the pseudo-reference sequence employing the SOAP software. Two mismatches were allowed on each strand of the PE read. SNPs of each DH individual were identified and sorted afterward to obtain the following information: the start and end sites of each PE read on the PRF, the number of SNPs on each read, the relative position of each SNP on the 200 bp unique tag, nucleotide variation and quality score for each SNP. The SNPs of a PE read were expressed as: 1201_1400 2 15@T- > C 30 196@T- > C 38, where 1201_1400 standed for the start and end sites of the PE read on the PRF, 2 standed for two SNPs existed on this read, 15 and 196 for the relative positions of the SNPs on the 200 bp unique tag, and 30 and 38 for the quality score of the two SNPs respectively. Next, all SNPs from the parents and all DH individuals were sorted according to the position of the sequence read on the PRF and the relative positions of SNPs on the 200 bp unique tag. The coverage depth and average quality of each SNP variation was counted in individual parents and DHs. All these processes in this section were performed by the prf_snpcalling.sh script and depicted in Figure 2B.

#### Discrimination of allelic SNPs from homoeologous loci

In oilseed rape, several types of mixed sequence reads from homoeologous loci are usually aligned to the same position on the pseudo-reference sequence. To mine allelic variants from mixed sequence reads (mixed alleles) at the same position, we first designated the alleles as A1, A2, A3 etc. The occurrence, coverage depth of each allele in each individual and the average quality score of each nucleotide variation were analyzed. The presence of an allele in a DH line was scored as “+”, and the absence of an allele in a DH line was scored as “-”. All alleles at a locus in the population were scored and tabulated in a matrix for genotype calling. The next step was to make a co-occurrence matrix and determined which two variants were allelic variations of a locus in the segregating DH population. Theoretically, all loci in DH lines are homozygous. If two alleles belong to a locus, only one allele rather than two alleles occur in a DH line simultaneously. If two alleles occur in the same DH individual simultaneously, they are definitely from two homoeologous loci. Based on this principle, allelic variants were discriminated for each locus using the script of prf_allele.sh (Figure 2C). The genotypes of a DH individual homozygous for alleles from ZY821 and No2127 were scored as “A” and “B” respectively, and missing data and heterozygous loci were scored as “0”.

To discover and genotype PAVs, only DH lines with about 2 million reads or more were selected to avoid false genotyping and sequence reads were aligned to the pseudo-reference sequence as described above. Only those loci that were covered by more than three reads and uniquely and perfectly matched to the pseudo-reference sequence were subjected to further analysis. The sequence reads that was absent in a DH and also in one of the parents were scored as null alleles.

### Construction of genetic linkage maps

All SNP loci with less than 25% MMD were used for linkage map construction. The MSTMap software was used to process the large dataset and construct a high-density genetic map [29]. All the genotypes of SNP loci and single-locus anchor SSR loci were formatted to meet the requirement of MSTMap. All these loci were first partitioned into LGs according to anchor SSR loci. The major parameters for loci partition were as follows: distance_function for kosambi; p_value for 0.0000001; no_map_dist for 10; missing_threshold for 0.25; objective_function for COUNT. The loci on each linkage group were ordered into bins separately. Then the missing genotypes were imputed using the argmax.geno algorithm in R/qtl package [42]. The parameters were as follows: step for 5, off.end for 5, error.prob for 0.05 and map.function for kosambi. After imputation, we reconstructed the genetic linkage bin map using MSTMap again with the same parameters.

In order to assign the dominant PAV markers to the 19 LGs of *B*. *napus*, at least one SNP loci with less than 10% MMD was selected from each bin from the SNP bin map. All putative dominant markers and the selected SNPs were submitted to map construction using MSTMap with the same parameters as described above.

### Validation of SNPs and genotypes

A suit of SNPs that create or destroy a restriction site was randomly selected for validation by CAPS markers. Primers were designed to amplify the target fragment including the SNP variations. PCR amplifications were performed in a volume of 20 μl containing about 100 ng DNA templates, 1× Taq buffer, 4 mM MgCl_2_, 0.4 mM dNTPs, 5 pmol each primer and 0.4 U Taq DNA polymerase (Fermentas). The reactions were carried out at 94°C for 3 min, 35 cycles of 94°C for 30 s, 60°C for 1 min, 72°C for 45 s, one final extension step of 72°C for 5 min, and kept at 4°C. After amplification, 5 μl PCR products were digested using 2 U corresponding RE and reaction buffer in a volume of 8 μl. The products were separated on 6% denaturing polyacrylamide gels and visualized using silver staining. The SNPs that were validated between the two parents were subjected to genotypes analysis in the DH population. Meanwhile, the PCR products of two parents were also directly sequenced to validate the variations.

### Data and software

The Illumina sequencing data from this study have been deposited in the NCBI Sequence Read Archive under accession number SRA056328. RFAPtools_v1.0 and pseudo-reference sequence constructed in this study “ZY821_prf.fasta” are available at http://211.69.128.139/~xunchen/RFAP/index.php.

## Abbreviations

AFLP: Amplified fragment length polymorphism; CRoPS: Complexity reduction of polymorphic sequences; ddRADseq: Double digest RADseq; ddGBS: Double digestion GBS; DH: Double haploid; GBS: Genotyping by sequencing; IHP: Inter-homoeolog polymorphism; LG: Linkage group; MMD: Maximum missing data; PAV: Presence/absence variation; PRF: Pseudo-reference sequence; RADseq: Restriction-site-associated DNA sequencing; RE: Restriction enzyme; RRL: Reduced representation library; SNP: Single nucleotide polymorphism; SSR: Simple sequence repeat

## Competing interests

The authors declare that they have no competing interests.

## Authors’ contributions

XC, XL, JX, ZW, BZ and HL performed the experiments. XC, YL and BW analyzed the data and performed the bioinformatics analyses. JW provided the plant materials. KL, XC and MY wrote the paper. KL, XC and SH conceived the study. The manuscript was read and approved by all authors.

## Supplementary Material

Additional file 1: Figure S1 Shows the modified ddRADseq protocol. **Figure S2.** Shows the number of sequence reads of the 91 DH lines. **Figure S3.** Shows the distribution of SNPs along pair-end sequence reads. **Figure S4.** Shows the PAV genetic map constructed with 45 DH individuals with about 2.0 million of sequence reads. **Figure S5.** Shows collinearity between the *B. rapa* and the A sub-genome in *B. napus* revealed by the PAV linkage groups. **Figure S6.** Shows four instances of mis-ordered sequence scaffolds on the *B. rapa* A10 chromosome. Click here for file

Additional file 2: Table S1 Shows the summary information of the PAV genetic linkage map. **Table S2.** Shows the information of possible misassembled sequence scaffolds detected by the SNP and PAV genetic linkage maps. **Table S3.** Shows the information of assignments of unassembled sequence scaffolds using the SNP and PAV genetic linkage maps. **Table S4.** Shows the sequences of adapters used in this study. Click here for file
