# Supplementary material for: Detection and genotyping of restriction fragment associated polymorphisms in polyploid crops with a pseudo-reference sequence: a case study in allotetraploid Brassica napus
Source: BMC Genomics. 2013 May 24;14:346. doi: 10.1186/1471-2164-14-346 (PMC3665465; doi:10.1186/1471-2164-14-346)
Supplement: Additional file 1: Figure S1 — Shows the modified ddRADseq protocol. Figure S2. Shows the number of sequence reads of the 91 DH lines. Figure S3. Shows the distribution of SNPs along pair-end sequence reads. Figure S4. Shows the PAV genetic map constructed with 45 DH individuals with about 2.0 million of sequence reads. Figure S5. Shows collinearity between the B. rapa and the A sub-genome in B. napus revealed by the PAV linkage groups. Figure S6. Shows four instances of mis-ordered sequence scaffolds on the B. rapa A10 chromosome. [file 1471-2164-14-346-S1.pdf]

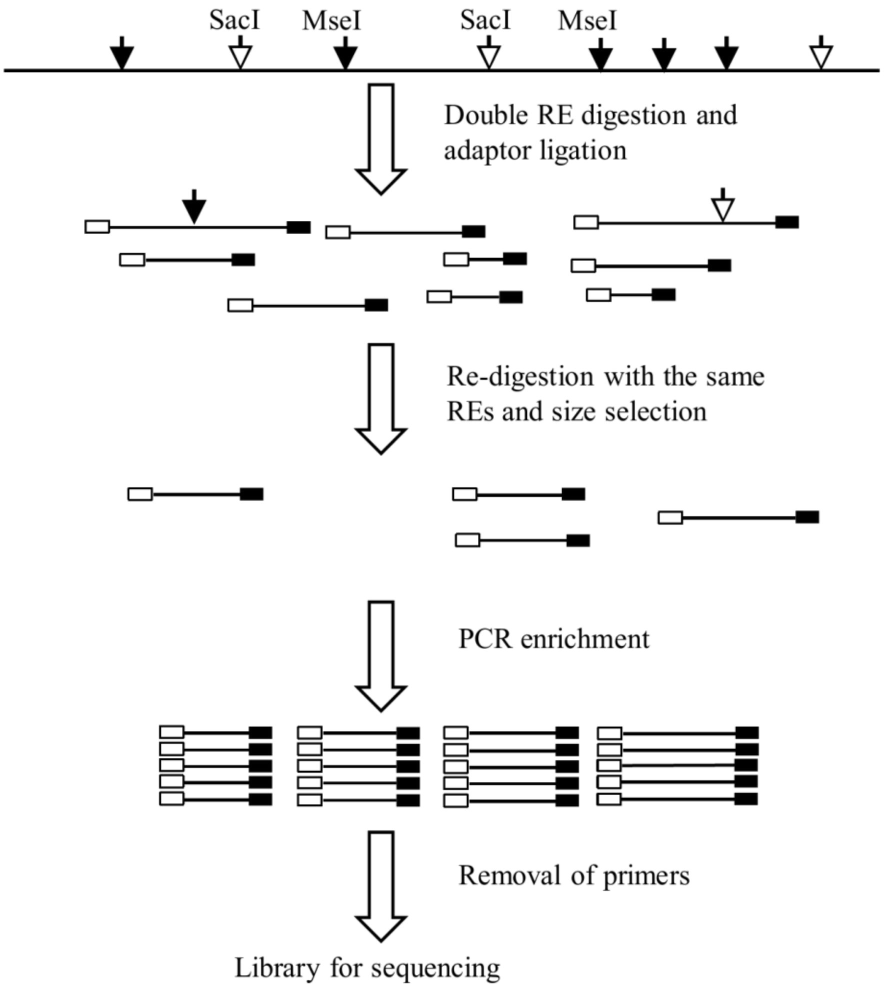


**Figure S1. Flowchart for modified ddRADseq sequencing library construction.**

The solid-headed and hollow-headed arrows indicate the SacI and MseI recognition site respectively. The horizontal lines represent the genomic sequence, and the solid and hollow boxes represent the Sac_AD and Mse_AD adaptor respectively.

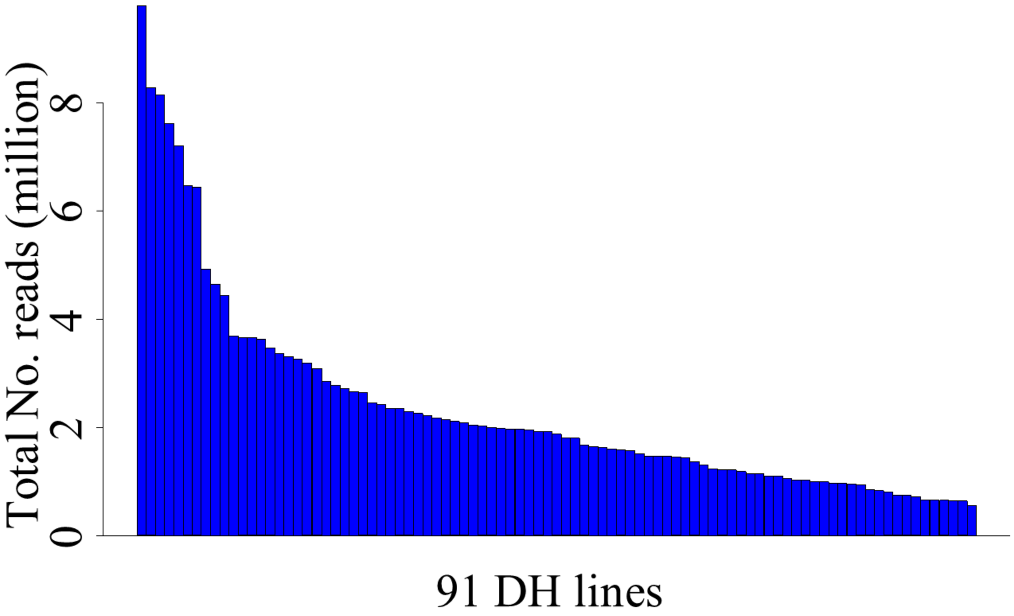


**Figure S2. The number of Sequence reads of the 91 DH lines.**

The lowest sequencing data is just 0.57 million reads, and the highest reach up to 9.79 million reads, with the average of 2.33 million reads.

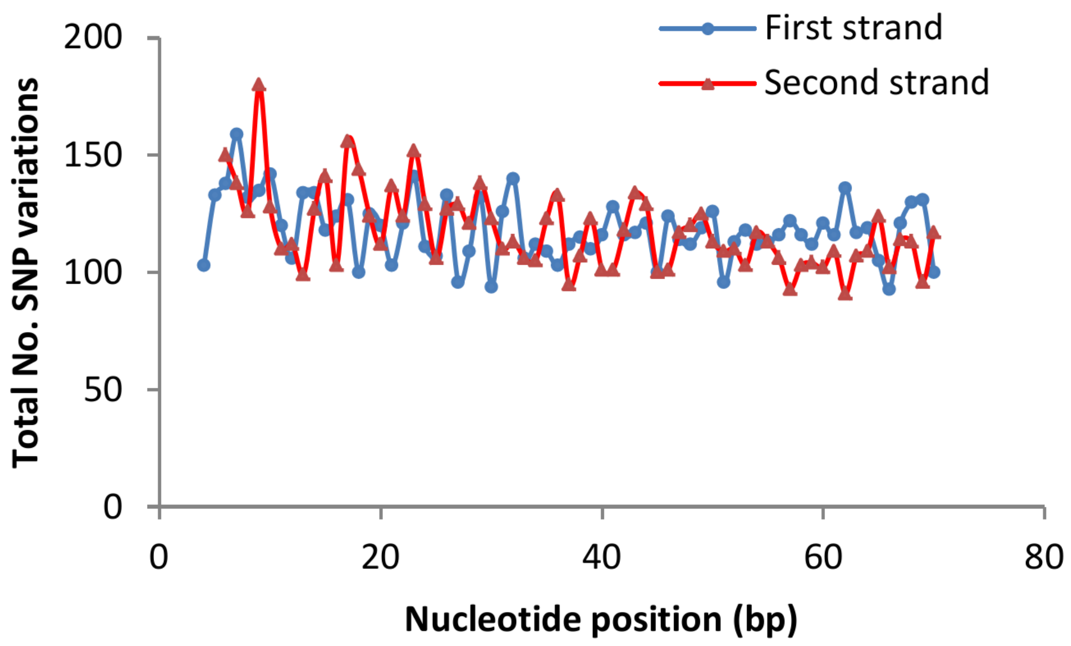


**Figure S3. The distribution of the total number of SNP variations on each nucleotide position of each pair-end read.**

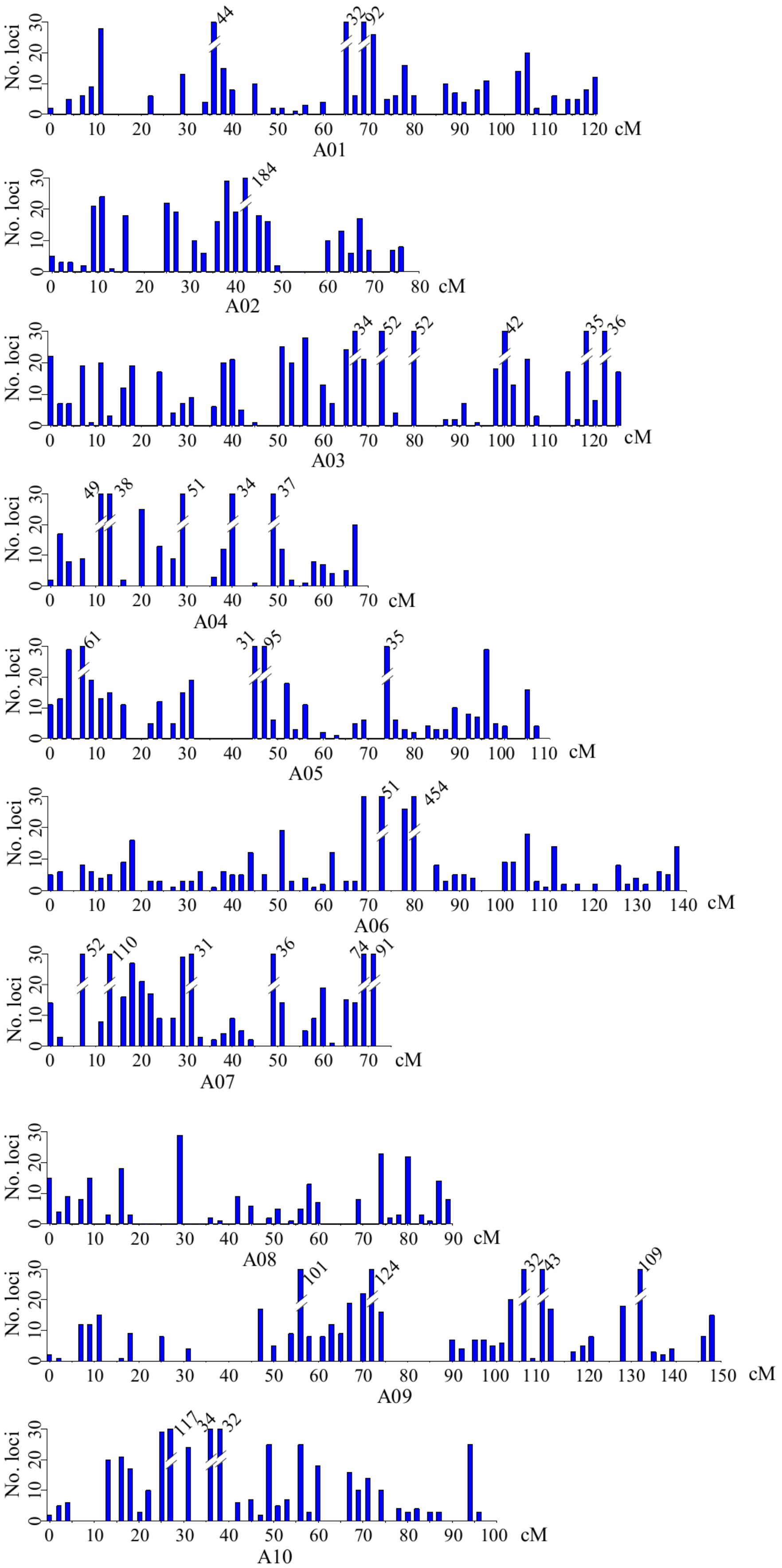
No. loci
cM
A01
44
32
92
A02
184
A03
34
52
52
42
35
36
A04
49
38
51
34
37
A05
61
31
95
35
A06
51
454
A07
52
110
31
36
74
91
A08
A09
101
124
32
43
109
A10
117
34
32

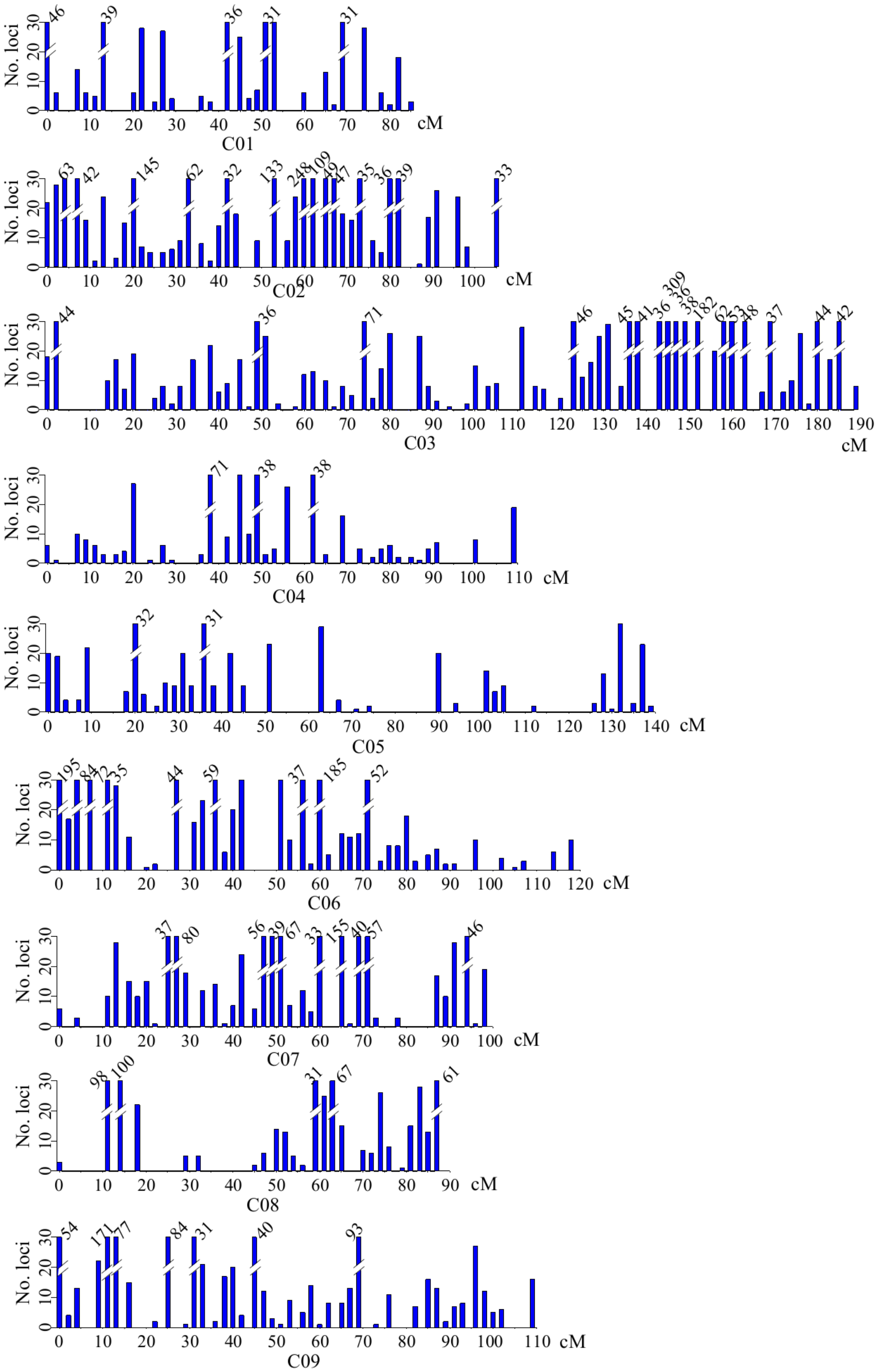


**Figure S4. The PAV genetic map constructed with 45 DH individuals with about 2.0 million of sequence reads.**

The X axis indicates the genetic distance (cM), and the height of bars represents the number of makers in each bin. The height of bars is limited under 30 in order to discriminate lower value. The values more than 30 are signed above the bars with corresponding numbers.

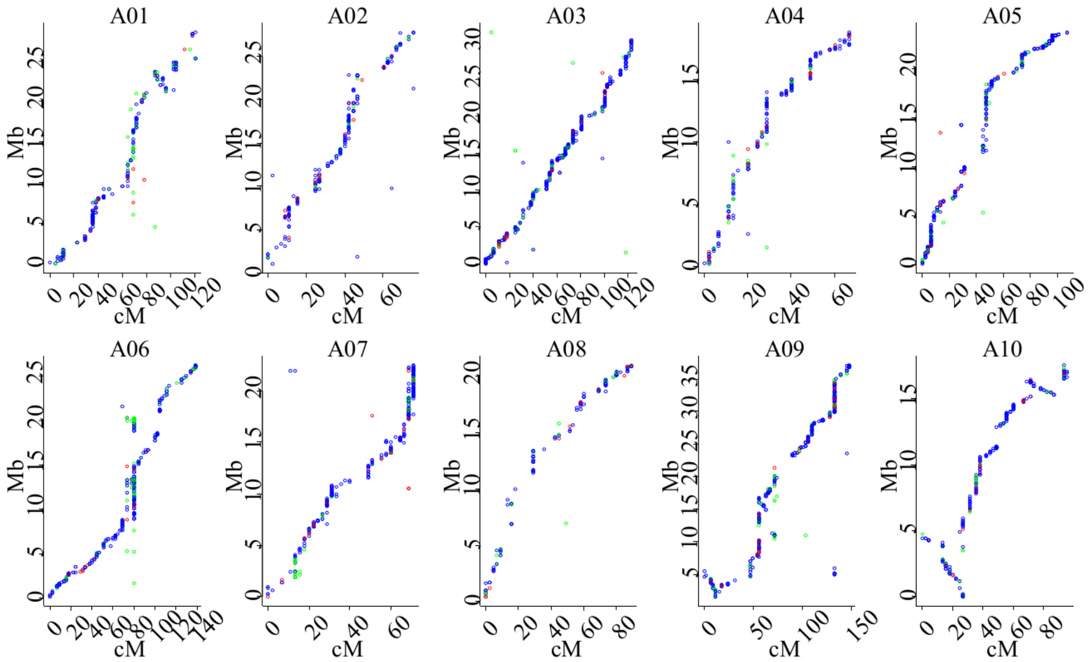


**Figure S5. Collinearity between the A genomes of *B. rapa* and *B. napus* revealed by the PAV linkage groups.**

The X axis represents the genetic distance in each linkage group of *B. napus*, and the Y axis represents the physical distance of corresponding *B. rapa* chromosome. If a locus is mapped to multiple paralogous positions in the *B. rapa* genome (green points), only the location with the best hit is selected here (E value <=1e-10). The red points represent loci with only one end of the PE read having a unique position in the *B. rapa* genome. The blue points represent loci with both ends of the PE read having a unique position in the *B. rapa* genome.

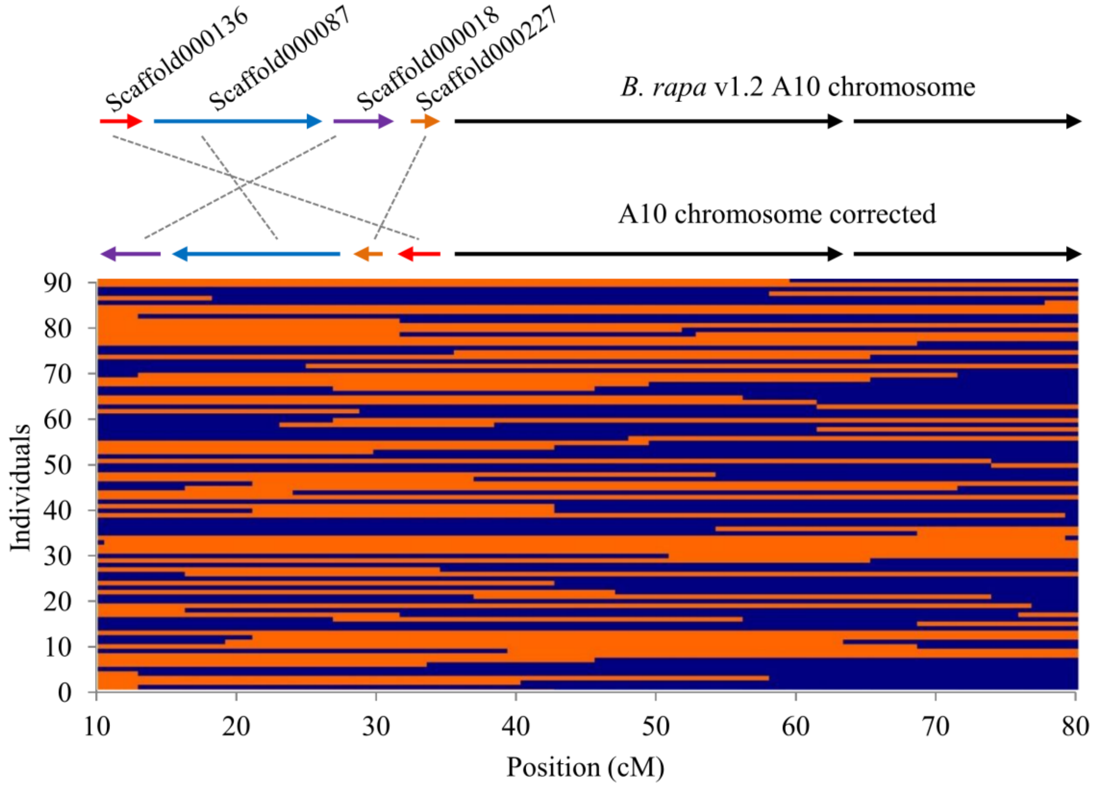


**Figure S6. The four instances of mis-ordered sequence scaffolds on the *B. rapa* A10 chromosome.**
