# Supplementary material for: Detection and genotyping of restriction fragment associated polymorphisms in polyploid crops with a pseudo-reference sequence: a case study in allotetraploid Brassica napus
Source: BMC Genomics. 2013 May 24;14:346. doi: 10.1186/1471-2164-14-346 (PMC3665465; doi:10.1186/1471-2164-14-346)
Supplement: Additional file 2: Table S1 — Shows the summary information of the PAV genetic linkage map. Table S2. Shows the information of possible misassembled sequence scaffolds detected by the SNP and PAV genetic linkage maps. Table S3. Shows the information of assignments of unassembled sequence scaffolds using the SNP and PAV genetic linkage maps. Table S4. Shows the sequences of adapters used in this study. [file 1471-2164-14-346-S2.pdf]

Table S1. Summary information of the PAV genetic linkage map

Linkage groups	Map length (cM)	No. bins	No. loci	Linkage groups	Map length (cM)	No. bins	No. loci
A01	120.4	38	463(37)	C01	84.7	28	434(34)
A02	75.9	26	486(35)	C02	104.6	42	1422(59)
A03	124.7	44	704(65)	C03	189.3	69	1758(140)
A04	66.8	24	369(28)	C04	109.3	35	390(26)
A05	107.1	38	545(41)	C05	139.1	35	422(64)
A06	138.0	53	846(58)	C07	98.0	36	886(44)
A07	71.1	29	649(43)	C06	118.1	41	1089(55)
A08	89.3	28	239(33)	C08	87.4	25	578(81)
A09	148.0	42	738(43)	C09	109.1	41	876(57)
A10	95.9	33	513(41)				
A genome	1037.2	355	5552(424)	C genome	1039.6	352	7855(560)

The number in the parenthesis represents the number of anchor SNP loci selected from the SNP bin map.

Table S2. Misassembled sequence scaffolds detected by the SNP and PAV genetic linkage maps.

B. rapa reference genome sequence						Sequence scaffolds corrected by the two linkage maps					
**Scafflods**	**Mis-orientation or misassembly**	**Chr**	**Start (bp)**	**End (bp)**	**Scaffold size (bp)**	**LG**	**SNP loci**	**PAV loci**	**Location on SNP genetic map (cM)**	**Target pos. start (bp)**	**Target pos. end (bp)**
**Scaffold000034**	misassembly	**A01**	25634329	26238368	604039	A01	12(2)	0	63.288	15488289	16279920
**Scaffold000025**	misassembly	**A05**	8425823	9268763	842940	A02	4(3)	11(7)	34.117	15807442	18684338
**Scaffold000101**	mis-orientation	**A07**	21718107	21786618	68511	A02	4(1)	5(1)	34.117	15807442	18684338
**Scaffold000009**	misassembly	**A06**	20093753	20821184	727431	A05	8(3)	0	25.365-26.465	12158062	16103501
**Scaffold000031**	misassembly	**A02**	23081296	23308915	227619	A05	6(5)	7(5)	70.482	23289456	23628827
**Scaffold000078**	misassembly	**A02**	26634175	26950142	315967	A05	6(5)	5(4)	74.88	23289456	23628827
**Scaffold000033**	misassembly	**A01**	24022993	24255919	232926	A06	14(3)	5(3)	69.382	12351689	14389970
**Scaffold000115**	misassembly	**A02**	22612117	23031115	418998	A06	5(4)	18(5)	69.382	12351689	14389970
**Scaffold000009**	misassembly	**A06**	18839422	20272409	1432987	A06	23(4)	33(13)	69.382	12351689	14389970
**Scaffold000002**	misassembly	**A10**	11473202	12726968	1253766	A07	18(15)	20(19)	5.501-9.897	887711	2061965
**Scaffold000062**	mis-orientation	**A02**	12493	1018781	1006288	A07	22(15)	16(15)	37.381-43.984	11015459	11764374
**Scaffold000138**	mis-orientation	**A08**	6675411	6716565	41154	A08	4(1)	0	10.993	8948858	11466227
**Scaffold000102**	mis-orientation	**A08**	9244877	9728370	483493	A09	6(6)	7(6)	39.835	17996638	18504784
**Scaffold000059**	misassembly	**A09**	19551247	19992305	441058	A09	5(5)	0	39.835	17996638	18504784
**Scaffold000145**	misassembly	**A09**	10941188	11251266	310078	A09	4(2)	6(5)	39.835	17996638	18504784
**Scaffold000135**	misassembly	**A09**	11338004	11435279	97275	A09	3(2)	0	39.835	17996638	18504784
**Scaffold000134**	misassembly	**A09**	10349068	10820428	471360	A09	6(3)	2(0)	47.530	17996638	23370803
**Scaffold000087**	mis-orientation	**A10**	3788898	4772390	983492	A10	7(6)	14(2)	0-5.506	0	4985709
**Scaffold000018**	mis-orientation	**A10**	482535	2848924	2366389	A10	30(22)	35(5)	5.506-16.505	0	4985709
**Scaffold000227**	mis-orientation	**A10**	4871003	4913983	42980	A10	2(1)	1(1)	16.505	0	4985709
**Scaffold000136**	mis-orientation	**A10**	4627	432451	427824	A10	13(6)	11(7)	17.604	0	4985709
**Scaffold000061**	misassembly	**A01**	18712242	19293378	581136	A10	9(6)	0	17.604	0	4985709

The number in the parenthesis indicates the number of loci uniquely mapped to one location in the *B. rapa* reference genome sequence.

Table S3. Assignments of unassembled sequence scaffolds using the SNP and PAV genetic linkage maps.

Scaffolds	Length (bp)	# loci	SNP genetic map			PAV genetic map		
			No. loci	LGs	Genetic distance (cM)	No. loci	LGs	Genetic distance (cM)
Scaffold000100	884,746	55	17	A06	66.084-69.382	38	A06	73.417-80.091
Scaffold000103	841,798	25	7	A02	34.117-35.216	18	A02	42.349
Scaffold000104	773,703	9	3	A04	14.302	6	A04	13.351
Scaffold000111	635,699	17	6	A02	34.117	11	A02	42.349
Scaffold000123	554,582	19	12	A10	16.505-17.604	7	A10	24.56-26.784
Scaffold000141	436,032	33	24	A06	69.382	9	A06	73.417-80.091
Scaffold000162	331,999	4	2	A03	123.274	2	A03	124.713
Scaffold000164	313729	13	3	A01	90.787-91.886	10	A01	104.81-113.714
Scaffold000167	296,181	5	3	A02	34.117	2	A02	42.349
Scaffold000169	287,768	14	11	A09	40.934-48.629	3	A06	69.555-73.417
Scaffold000170	283,223	14				14	A10	24.560-26.784
Scaffold000172	276,837	5	2	A05	24.266	3	A05	44.831
Scaffold000180	225,530	3	1	A02	34.117	2	A02	42.349
Scaffold000185	212,102	13	3	A09	47.530	10	A09	71.779-74.002
Scaffold000191	177,795	7	3	A05	73.781-74.880	4	A05	104.906
Scaffold000193	152,059	4	1	A10	16.505	3	A10	24.560
Scaffold000196	138,308	4	4	A10	16.505			
Scaffold000203	109,728	2	1	A05	71.582	1	A05	104.906
Scaffold000207	98,578	3				3	A02	42.349
Scaffold000208	98,184	10	7	A06	68.283-69.382	3	A06	80.091
Scaffold000211	90,472	3	3	A10	17.604			
Scaffold000212	88,452	6	6	A10	17.604			
Scaffold000213	88,292	3				3	A02	42.349
Scaffold000218	78,497	3	3	A05	20.965			
Scaffold000233	65,372	4	2	A02	34.117	2	A02	42.349
Scaffold000234	65,355	2				2	A07	13.360
Scaffold000238	58,889	3	1	A10	16.505	2	A10	24.560

Scaffold000239	57,644	3	1	A07	10.996	2	A07	13.360
Scaffold000247	52,366	3				3	A08	15.575-17.798
Scaffold000261	45,658	2				2	A05	31.167
Scaffold000263	45,307	2				2	A03	124.713
Scaffold000273	39,897	2				2	A06	80.091
Scaffold000286	34,788	4	2	A10	17.604	2	A10	26.784
Scaffold000311	28,736	2	2	A06	69.382			
Scaffold000320	26,556	3	2	A08	28.896	1	A08	29.098
Scaffold000328	25,411	2				2	A07	13.360
Scaffold000341	21,996	2	2	A09	47.530			
Scaffold000357	20,520	2	1	A10	17.604	1	A10	26.784
Scaffold000362	19,990	2	2	A09	38.735			
Scaffold000416	13,477	4	1	A06	69.382	3	A06	80.091
Scaffold000449	11,426	2	1	A10	17.604	1	A10	26.784
Scaffold000460	10,964	2				2	A09	105.657
Scaffold000553	5,809	2				2	A07	13.360
Scaffold000618	3,901	2				2	A06	80.091

Table S4. Sequences of adapters used in this study.

Adapters	Sequences
Sac01-T	5'-ACACTCTTTCCCTACACGACGCTCTTCCGATCTGCATAAGCT
Sac01-B	5'p-TATGCAGATCGGAAGAGCGTCGTGTAGGGAAAGAGTGT
Sac02-T	5'-ACACTCTTTCCCTACACGACGCTCTTCCGATCTGTACAAGCT
Sac02-B	5'p-TGTACAGATCGGAAGAGCGTCGTGTAGGGAAAGAGTGT
Sac03-T	5'-ACACTCTTTCCCTACACGACGCTCTTCCGATCTACTGAAGCT
Sac03-B	5'p-TCAGTAGATCGGAAGAGCGTCGTGTAGGGAAAGAGTGT
Sac04-T	5'-ACACTCTTTCCCTACACGACGCTCTTCCGATCTTACGAAGCT
Sac04-B	5'p-TCGTAAGATCGGAAGAGCGTCGTGTAGGGAAAGAGTGT
Sac05-T	5'-ACACTCTTTCCCTACACGACGCTCTTCCGATCTCAGACAGCT
Sac05-B	5'p-GTCTGAGATCGGAAGAGCGTCGTGTAGGGAAAGAGTGT
Sac06-T	5'-ACACTCTTTCCCTACACGACGCTCTTCCGATCTCGATCAGCT
Sac06-B	5'p-GATCGAGATCGGAAGAGCGTCGTGTAGGGAAAGAGTGT
Sac07-T	5'-ACACTCTTTCCCTACACGACGCTCTTCCGATCTTTAGCAGCT
Sac07-B	5'p-GCTAAAGATCGGAAGAGCGTCGTGTAGGGAAAGAGTGT
Sac08-T	5'-ACACTCTTTCCCTACACGACGCTCTTCCGATCTATCGCAGCT
Sac08-B	5'p-GCGATAGATCGGAAGAGCGTCGTGTAGGGAAAGAGTGT
Sac09-T	5'-ACACTCTTTCCCTACACGACGCTCTTCCGATCTCGTATAGCT
Sac09-B	5'p-ATACGAGATCGGAAGAGCGTCGTGTAGGGAAAGAGTGT
Sac10-T	5'-ACACTCTTTCCCTACACGACGCTCTTCCGATCTGCCATAGCT
Sac10-B	5'p-ATGGCAGATCGGAAGAGCGTCGTGTAGGGAAAGAGTGT
Sac11-T	5'-ACACTCTTTCCCTACACGACGCTCTTCCGATCTAGACTAGCT
Sac11-B	5'p-AGTCTAGATCGGAAGAGCGTCGTGTAGGGAAAGAGTGT
Sac12-T	5'-ACACTCTTTCCCTACACGACGCTCTTCCGATCTTAGCTAGCT
Sac12-B	5'p-AGCTAAGATCGGAAGAGCGTCGTGTAGGGAAAGAGTGT
Sac13-T	5'-ACACTCTTTCCCTACACGACGCTCTTCCGATCTAGCTAAGCT
Sac13-B	5'p-TAGCTAGATCGGAAGAGCGTCGTGTAGGGAAAGAGTGT
Sac14-T	5'-ACACTCTTTCCCTACACGACGCTCTTCCGATCTCAGTAAGCT
Sac14-B	5'p-TACTGAGATCGGAAGAGCGTCGTGTAGGGAAAGAGTGT
Sac15-T	5'-ACACTCTTTCCCTACACGACGCTCTTCCGATCTCGTCAAGCT
Sac15-B	5'p-TGACGAGATCGGAAGAGCGTCGTGTAGGGAAAGAGTGT
Sac16-T	5'-ACACTCTTTCCCTACACGACGCTCTTCCGATCTATGCAAGCT
Sac16-B	5'p-TGCATAGATCGGAAGAGCGTCGTGTAGGGAAAGAGTGT
Sac17-T	5'-ACACTCTTTCCCTACACGACGCTCTTCCGATCTGCTACAGCT
Sac17-B	5'p-GTAGCAGATCGGAAGAGCGTCGTGTAGGGAAAGAGTGT
Sac18-T	5'-ACACTCTTTCCCTACACGACGCTCTTCCGATCTTGCACAGCT
Sac18-B	5'p-GTGCAAGATCGGAAGAGCGTCGTGTAGGGAAAGAGTGT
Sac19-T	5'-ACACTCTTTCCCTACACGACGCTCTTCCGATCTACGTCAGCT
Sac19-B	5'p-GACGTAGATCGGAAGAGCGTCGTGTAGGGAAAGAGTGT
Sac20-T	5'-ACACTCTTTCCCTACACGACGCTCTTCCGATCTAATGCAGCT
Sac20-B	5'p-GCATTAGATCGGAAGAGCGTCGTGTAGGGAAAGAGTGT
Sac21-T	5'-ACACTCTTTCCCTACACGACGCTCTTCCGATCTCTGATAGCT
Sac21-B	5'p-ATCAGAGATCGGAAGAGCGTCGTGTAGGGAAAGAGTGT

Sac22-T	5'-ACACTCTTTCCCTACACGACGCTCTTCCGATCTGACTTAGCT
Sac22-B	5'p-AAGTCAGATCGGAAGAGCGTCGTGTAGGGAAAGAGTGT
Sac23-T	5'-ACACTCTTTCCCTACACGACGCTCTTCCGATCTGATCTAGCT
Sac23-B	5'p-AGATCAGATCGGAAGAGCGTCGTGTAGGGAAAGAGTGT
Sac24-T	5'-ACACTCTTTCCCTACACGACGCTCTTCCGATCTTCAGTAGCT
Sac24-B	5'p-ACTGAAGATCGGAAGAGCGTCGTGTAGGGAAAGAGTGT
Mse01-T	5'p-TATATGCAGATCGGAAGAGCGGTTCAGCAGGAATGCCGAG
Mse01-B	5'-CTCGGCATTCCTGCTGAACCGCTCTTCCGATCTGCATA
Mse02-T	5'p-TATGTACAGATCGGAAGAGCGGTTCAGCAGGAATGCCGAG
Mse02-B	5'-CTCGGCATTCCTGCTGAACCGCTCTTCCGATCTGTACA
Mse03-T	5'p-TATCAGTAGATCGGAAGAGCGGTTCAGCAGGAATGCCGAG
Mse03-B	5'-CTCGGCATTCCTGCTGAACCGCTCTTCCGATCTACTGA
Mse04-T	5'p-TATCGTAAGATCGGAAGAGCGGTTCAGCAGGAATGCCGAG
Mse04-B	5'-CTCGGCATTCCTGCTGAACCGCTCTTCCGATCTTACGA
Mse05-T	5'p-TAGTCTGAGATCGGAAGAGCGGTTCAGCAGGAATGCCGAG
Mse05-B	5'-CTCGGCATTCCTGCTGAACCGCTCTTCCGATCTCAGAC
Mse06-T	5'p-TAGATCGAGATCGGAAGAGCGGTTCAGCAGGAATGCCGAG
Mse06-B	5'-CTCGGCATTCCTGCTGAACCGCTCTTCCGATCTCGATC
Mse07-T	5'p-TAGCTAAAGATCGGAAGAGCGGTTCAGCAGGAATGCCGAG
Mse07-B	5'-CTCGGCATTCCTGCTGAACCGCTCTTCCGATCTTTAGC
Mse08-T	5'p-TAGCGATAGATCGGAAGAGCGGTTCAGCAGGAATGCCGAG
Mse08-B	5'-CTCGGCATTCCTGCTGAACCGCTCTTCCGATCTATCGC
Mse09-T	5'p-TACTACGAGATCGGAAGAGCGGTTCAGCAGGAATGCCGAG
Mse09-B	5'-CTCGGCATTCCTGCTGAACCGCTCTTCCGATCTCGTAG
Mse10-T	5'p-TACTGGCAGATCGGAAGAGCGGTTCAGCAGGAATGCCGAG
Mse10-B	5'-CTCGGCATTCCTGCTGAACCGCTCTTCCGATCTGCCAG
Mse11-T	5'p-TACGTCTAGATCGGAAGAGCGGTTCAGCAGGAATGCCGAG
Mse11-B	5'-CTCGGCATTCCTGCTGAACCGCTCTTCCGATCTAGACG
Mse12-T	5'p-TACGCTAAGATCGGAAGAGCGGTTCAGCAGGAATGCCGAG
Mse12-B	5'-CTCGGCATTCCTGCTGAACCGCTCTTCCGATCTTAGCG
Mse13-T	5'p-TATAGCTAGATCGGAAGAGCGGTTCAGCAGGAATGCCGAG
Mse13-B	5'-CTCGGCATTCCTGCTGAACCGCTCTTCCGATCTAGCTA
Mse14-T	5'p-TATACTGAGATCGGAAGAGCGGTTCAGCAGGAATGCCGAG
Mse14-B	5'-CTCGGCATTCCTGCTGAACCGCTCTTCCGATCTCAGTA
Mse15-T	5'p-TATGACGAGATCGGAAGAGCGGTTCAGCAGGAATGCCGAG
Mse15-B	5'-CTCGGCATTCCTGCTGAACCGCTCTTCCGATCTCGTCA
Mse16-T	5'p-TATGCATAGATCGGAAGAGCGGTTCAGCAGGAATGCCGAG
Mse16-B	5'-CTCGGCATTCCTGCTGAACCGCTCTTCCGATCTATGCA
Mse17-T	5'p-TAGTAGCAGATCGGAAGAGCGGTTCAGCAGGAATGCCGAG
Mse17-B	5'-CTCGGCATTCCTGCTGAACCGCTCTTCCGATCTGCTAC
Mse18-T	5'p-TAGTGCAAGATCGGAAGAGCGGTTCAGCAGGAATGCCGAG
Mse18-B	5'-CTCGGCATTCCTGCTGAACCGCTCTTCCGATCTTGCAC
Mse19-T	5'p-TAGACGTAGATCGGAAGAGCGGTTCAGCAGGAATGCCGAG
Mse19-B	5'-CTCGGCATTCCTGCTGAACCGCTCTTCCGATCTACGTC

Mse20-T	5'p-TAGCATTAGATCGGAAGAGCGGTTCAGCAGGAATGCCGAG
Mse20-B	5'-CTCGGCATTCCTGCTGAACCGCTCTTCCGATCTAATGC
Mse21-T	5'p-TACTCAGAGATCGGAAGAGCGGTTCAGCAGGAATGCCGAG
Mse21-B	5'-CTCGGCATTCCTGCTGAACCGCTCTTCCGATCTCTGAG
Mse22-T	5'p-TACAGTCAGATCGGAAGAGCGGTTCAGCAGGAATGCCGAG
Mse22-B	5'-CTCGGCATTCCTGCTGAACCGCTCTTCCGATCTGACTG
Mse23-T	5'p-TACGATCAGATCGGAAGAGCGGTTCAGCAGGAATGCCGAG
Mse23-B	5'-CTCGGCATTCCTGCTGAACCGCTCTTCCGATCTGATCG
Mse24-T	5'p-TACCTGAAGATCGGAAGAGCGGTTCAGCAGGAATGCCGAG
Mse24-B	5'-CTCGGCATTCCTGCTGAACCGCTCTTCCGATCTTCAGG

Red letters stand for the barcode sequences, and the "p" at the end of sequence means that the nucleotide is 5'-phosphorylated.
